# Hemodynamic impact of early mobilization in critical patients receiving vasoactive drugs: A prospective cohort study

**DOI:** 10.1371/journal.pone.0279269

**Published:** 2022-12-20

**Authors:** Larissa Faria Borges, Renato Fraga Righetti, Davi de Souza Francisco, Wellington Pereira Yamaguti, Cassia Fabiane De Barros

**Affiliations:** Rehabilitation Service, Hospital Sírio-Libanês, São Paulo (SP), Brazil; Clinica Luganese Moncucco, SWITZERLAND

## Abstract

**Background:**

Vasoactive drugs are one of the most common patient-related barriers to early mobilization. Little is known about the hemodynamic effects of early mobilization on patients receiving vasoactive drugs. This study aims to observe and describe the impact of mobilization on the vital signs of critical patients receiving vasoactive drugs as well as the occurrence of adverse events.

**Methods:**

This is a cohort study performed in an Intensive Care Unit with patients receiving vasoactive drugs. All patients, either mobilized or non-mobilized, had their clinical data such as vital signs [heart rate, respiratory rate, systolic blood pressure, diastolic blood pressure, mean arterial pressure, and oxygen saturation], type and dosage of the vasoactive drug, and respiratory support collected at rest. For mobilized patients, the vital signs were also collected after mobilization, and so was the highest level of mobility achieved and the occurrence of adverse events. The criteria involved in the decision of mobilizing the patients were registered.

**Results:**

53 patients were included in this study and 222 physiotherapy sessions were monitored. In most of the sessions (n = 150, 67.6%), patients were mobilized despite the use of vasoactive drugs. There was a statistically significant increase in heart rate and respiratory rate after mobilization when compared to rest (p<0.05). Only two (1.3%) out of 150 mobilizations presented an adverse event. Most of the time, non-mobilizations were justified by the existence of a clinical contraindication (n = 61, 84.7%).

**Conclusions:**

The alterations observed in the vital signs of mobilized patients may have reflected physiological adjustments of patients’ cardiovascular and respiratory systems to the increase in physical demand imposed by the early mobilization. The adverse events were rare, not serious, and reversed through actions such as a minimal increase of the vasoactive drug dosage.

## Introduction

Early mobilization refers to any form of physical activity started as soon as possible with critically ill patients hospitalized in an Intensive Care Unit (ICU) [1, 2]. It is the main component for the prevention and treatment of ICU-acquired weakness [3, 4] and is associated with better short- and long-term patient prognoses [3, 5]. Previous studies have shown that early mobilization reduces the rates of functional capacity loss [2, 3, 6], *delirium* [7], rehospitalization [8], and mortality after hospital discharge [8, 9]. Despite the known benefits, safety, and feasibility of early mobilization [2, 10, 11], prolonged bed rest is still prevalent in Intensive Care Units [12, 13]. Structural, process, cultural, and patient-related barriers hinder the mobilization of critically ill patients [12, 14, 15] mainly when they are receiving vasoactive drugs [12, 16, 17], which have been described as the most common patient-related barrier of early mobilization [12].

Vasoactive drugs are inotropic, vasopressor, or vasodilator agents that provide temporary hemodynamic support to assist in the recovery of hemodynamically unstable patients [18]. Critically ill patients are already at risk of a broad spectrum of deleterious effects of immobilization [19]. Furthermore, in a randomized clinical trial, Wolfe *et al*. have shown that the use of vasoactive drugs is an independent risk factor for the development of ICU-acquired weakness, which highlights the importance of early mobilizing these patients [20]. To the best of our knowledge, little is known about the hemodynamic effects of early mobilization in patients receiving vasoactive drugs [1, 17, 21–24]. A current Systematic Review of five studies has shown that although mobilizing these patients appears to be safe in most cases, more prospective studies are needed to fill in some gaps regarding this practice [1].

The lack of studies regarding the hemodynamic impact of early mobilization in patients with vasoactive drugs enforces the barriers to its practice and justifies the need for scientific evidence to guarantee its execution with quality and safety and its insertion in the ICU’s routine for the benefit of the patients. Therefore, this study aims to observe and describe the impact of mobilization on the vital signs of critical patients receiving vasoactive drugs as well as the occurrence of adverse events.

## Methods

### Study design, settings, and ethical aspects

This is a prospective cohort study carried out in the Intensive Care Units of the *Hospital Sírio-Libanês* from July to September of 2019. This study was approved by the Human Research Ethics Committee of the institution (3.384.738 / CAAE 14436019.0.0000.5461).

### Eligibility criteria and selection of participants

The sample of this study consisted of patients hospitalized in the ICU, who were ≥ 18 years old, have signed the informed consent form or had it signed by a legal representative, and had been using, for at least two hours, one or more inotropic and/or vasopressor drug, without concomitant use of a vasodilator drug (e.g. sodium nitroprusside and nitroglycerin) [25]. Regarding the different actions of the vasodilators [25, 26] (2,3) when compared to inotropic/vasopressor [27] drugs and the different clinical profiles of the patients for whom they are medically indicated, the behavior of vital signs and the profile of adverse events might also differ in both cases. Therefore, to unify the sample and to provide a more assertive analysis, patients receiving vasodilators were not included in this study.

Other inclusion criteria were the absence of mechanical circulatory assist devices [e.g. intra-aortic balloon and extracorporeal membrane oxygenation (ECMO)] and the absence of intracranial hypertension and dysautonomia. The exclusion criteria were the withdrawal of the participant or the responsible family member from authorizing participation in this study at any time, and/or a change in clinical status after the inclusion in this study with limitation of end-of-life care and no escalation of the vasoactive drug.

The screening of participants was carried out daily by the main researcher through an active search in the electronic medical records. Patients who met the eligibility criteria were personally invited to participate in the study. After understanding the data collection procedures and clarifying possible doubts, the patients or the responsible family member (in case of patients without an adequate level of awareness) signed the consent form.

### Data collection

A collection form (S1 File) designed for this research was filled out at the bedside during physiotherapy. Data collection was carried out in morning and afternoon shifts. Night physiotherapy sessions were not included in this study since early mobilization is not a focus of this shift in the studied service.

The physiotherapist in charge of the patient on each shift was responsible for filling out the collection form at the time of the session. To guarantee the standardization of the filled-out forms, all the physiotherapists were instructed before the beginning of data collection and regular inspections of the filled forms were carried out by the main researcher. Furthermore, the professionals were trained to check the correct placement and the reliability of the signal of the monitoring devices (e.g.: pulse oximeter, chest electrodes, invasive and non-invasive arterial blood pressure devices) to ensure an accurate assessment of the vital signs.

Patients were connected to a monitor displaying vital signs on a 24-hour basis, as mandatory in an ICU environment. The physiotherapists collected the needed information through the patient’s anamnesis and continuous observation of the patient and the monitor during the physiotherapy sessions.

The demographics (age and sex), the primary cause of ICU admission, the type, and dosage of the vasoactive drug, the SOFA (Sequential Organ Failure Assessment), and the APACHE II (Acute Physiology and Chronic Health Evaluation) scores at the time of admission were collected for all patients included in this study—either mobilized or non-mobilized -, and so were the vital signs at rest [heart rate (HR), respiratory rate (RR), systolic blood pressure (SBP), diastolic blood pressure (DBP), mean arterial pressure (MAP), and oxygen saturation (SpO_2_)]. The use of sedation and respiratory support (oxygen therapy, high flow nasal cannula, non-invasive, and invasive mechanical ventilation) were also registered when applicable.

After collecting the initial data, the physiotherapists decided to mobilize or not mobilize the patients based on the evaluation of laboratory tests, the assessment of the patients, and the discussion of the indication of mobilization with the multidisciplinary team. The studied institution implemented an early mobilization guideline in 2011, which guides the physiotherapists towards the early mobilization practice as it establishes criteria of clinical instability (e.g. hemoglobin < 7g/dL, temperature > 38°C, use of accessory muscles of ventilation, presence of limiting symptoms of pain or fatigue, etc) to help constructing the rationale behind the decision making of mobilizing a patient or maintaining bed rest [28].

For non-mobilized patients, a justification for the rest was registered. For mobilized patients, the vital signs were collected once again, after the end of the physiotherapy, as well as the type and dosage of the vasoactive drug, and the respiratory support. The monitorization of vital signs was also carried throughout the mobilization, as patients were continuously assessed by the physiotherapist to guarantee the safety of the intervention and the screening of adverse events.

Data collection was immediately interrupted when patients were discharged from the ICU or when the use of vasoactive drugs stopped.

Electronic medical records of all patients included in this study were audited after the collection to obtain necessary information for cases of incompletely filled collection forms. Incomplete forms for which data could not be recovered through the audits were not included in this study.

### ICU Mobility Scale

The highest mobility milestone achieved during physiotherapy was classified according to the ICU Mobility Scale (IMS), a scale developed by Hodgson *et al*. [29] and translated and validated to Portuguese by Kawaguchi *et al*. [30]. The IMS is an easily applicable tool at the bedside, with strong inter-rater reliability and a high validity for measuring the maximum level of mobility of adult patients in the ICU [29, 31]. It categorizes the level of mobility of the patients according to the mobility milestones reached and the level of assistance needed [29, 30]. The scores of the IMS range on a scale from 0 to 10, in which the score of 0 is the lowest mobility level (patient passively mobilized in bed) and the score of 10 is the highest mobility level (patient walking for at least 5 meters without any assistance) [14, 29, 30].

There are a few validated scales developed to assess the function and/or mobility of ICU patients which have published clinimetric data [32]. The IMS was the chosen validated scale for this study because it was already part of the studied institution’s care model, well known by the professionals, and used daily to score patients’ mobility [33]. The familiarity of the physiotherapists with the scale would strengthen the data collection, reducing the chances of failures in patients’ mobility assessment through the tool. To better characterize the profile of the mobilizations performed in this study, we stratified the intensity of our mobilizations using the classification proposed by Rebel *et al*. [21], in which levels 1 and 2 correspond to low intensity, levels 3 to 5: moderate intensity, and levels 6 to 10: high intensity. Although passive mobilization in bed (IMS level 0) does not fit the most current definitions of early mobilization [2, 21], mobilizations at this level were recorded and analyzed in this study, since they can also present hemodynamic impact in critical patients [34].

### Adverse events

The incidence of adverse events related to the early mobilization of the patients was identified by the physiotherapists and documented in the collection form and also in the electronic medical records. In case of any adverse event during mobilization, the therapy was immediately stopped and the event reported, as well as any intervention needed for the patient’s stabilization after the event. Another mobilization attempt was performed on the next shift if indicated.

Adverse events were defined before data collection as hypotension leading to vasoactive drug escalation (as decided by the medical team, rather than an absolute threshold), new onset of cardiac arrhythmia, desaturation (SpO_2_ < 90%), the onset of respiratory distress signs, syncope, fall or near-fall of the patient, accidental removal of invasive devices (e.g. endotracheal tube, central or peripheral catheters, drains, urinary catheters, gastrostomy, and nasoenteral tube), and death [17, 21, 22, 35]. For adverse events resulting in changes in vital signs, we considered those which occurred during or until 10 minutes after the mobilization, to guarantee that they were truly related to the impact of the mobilization on the patient’s hemodynamics and not to any other possible decompensation of critically ill patients [36].

### Outcomes

The outcomes were: (A) the impact of mobilization on vital signs (HR, RR, SBP, DBP, MAP, and SpO_2_) and vasoactive drug dosage; (B) the highest mobility milestone achieved; and (C) the occurrence of adverse events.

Information on the vasoactive drug use was extracted from continuous infusion pumps and the vital signs were registered according to the observation of real-time monitors. In cases of non-invasive arterial pressure measurement, the MAP was automatically calculated by the electronic sphygmomanometer available in each ICU room.

### Statistical analysis

Data analysis was performed using GraphPad Prism (software version 8.0.0 for Windows, San Diego, California, United States of America). Categorical variables were represented by absolute and relative frequency. For all statistical tests, p<0.05 was considered statistically significant. The normality of the distribution of continuous variables was evaluated through the Shapiro-Wilk test and they were later represented by median and 25th and 75th percentiles (non-parametric data). The Wilcoxon test was used for the comparison of vital signs and vasoactive drugs dosage pre- and post-mobilization. Pearson’s correlation was used to assess the correlation between the level of mobility achieved during physiotherapy and the vasoactive drug dosage. Chi-Square was used for the comparison of clinical support needs during mobilizations and non-mobilizations. Sample calculation was based on the study performed by Rebel *et al*. [21], who found a significant difference in systolic blood pressure values obtained before and after early mobilization. Estimating to observe a similar effect and using a 5% error (test power = 80%), it was necessary to include 29 subjects in this study.

## Results

**Fig 1:**
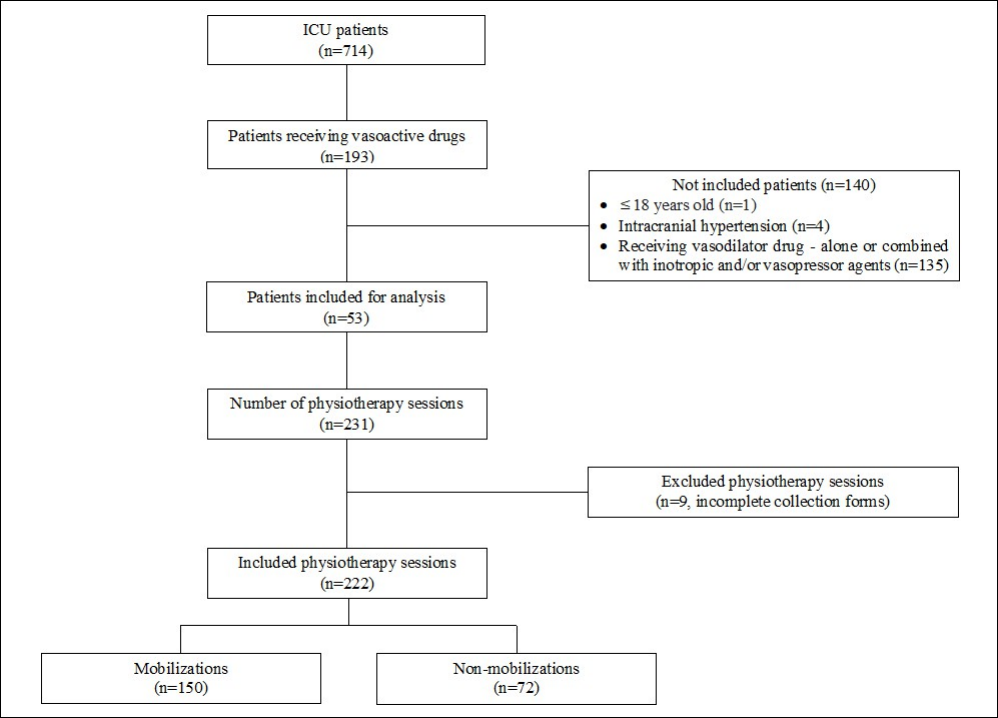
Flowchart of patient’s eligibility and physiotherapy sessions.

Overall, 53 individuals were included in this study (Fig 1). A total of 231 collection forms were filled out during physiotherapy sessions over 3 months. Nine incomplete forms were excluded from the study, resulting in 222 analyzed forms. Patients were mobilized in most of the physiotherapy sessions (n = 150, 67.6%).

### Sample’s demographics

Most of the subjects were male (n = 32, 60.4%) and the mean age of participants was 70.3 ± 16.7 years old. Surgical (n = 27, 50.9%) and medical (n = 26, 49.1%) causes of ICU admission presented a similar frequency. Sepsis (n = 11, 20.8%) was the most frequent clinical cause of ICU admission. Among the surgical causes, elective (n = 17, 32%) and urgent (n = 10, 18.9%) procedures were observed (Table 1).

**Table 1:** Demographic profile of the sample.

Characteristics	Values
Total patients, n (%)	53 (100)
Male, n (%)	32 (60.4)
Female, n (%)	21 (39.6)
Age, Mean ± SD	70.3 ± 16.7
Primary cause of ICU admission, n (%)	
*Surgical*	27 (50.9)
• Elective	17 (32)
• Urgent	10 (18.9)
*Medical*	26(49.1)
• Sepsis	11 (20.8)
• Cardiovascular	4 (7.5)
• Neurological	3 (5.7)
• Respiratory Failure	2(3.8)
• Others	6 (11.3)
APACHE II Score, Mean ± SD	15.75 ± 6.07
SOFA Score, Mean ± SD	5.18 ± 2.34

SD: standard deviation; ICU: Intensive Care Unit; APACHE II: Acute Physiology and Chronic Health Evaluation; SOFA: Sequential Organ Failure Assessment.

### Clinical support needs during mobilizations and non-mobilizations

The vasoactive drugs in use during physiotherapy sessions were Noradrenaline (n = 198, 89.2%), Dobutamine (n = 51, 23%), Vasopressin (n = 12, 5.4%) and Milrinone (n = 7, 3.2%). The concomitant use of two or more vasoactive drugs was computed for all the drugs involved and, therefore, the relative frequencies of the vasoactive drugs exceed 100% (Table 2).

**Table 2:** Clinical support needs during mobilizations and non-mobilizations.

	Total (n = 222)		Mobilizations (n = 150)		Non-mobilizations (n = 72)		
	n	%	n	%	n	%	P value
Vasoactive Drug							
Noradrenaline	198	89.2	132	88	66	91.7	NS
Dobutamine	51	23	39	26	12	16.7	NS
Vasopressin	12	5.4	5	3.3	7	9.7	NS
Milrinone	7	3.2	5	3.3	2	2.8	NS
Combined drugs	45	20.3	33	22	12	16.7	NS
Sedation	26	11.7	11	7.3	15	20.8	0.03*
Respiratory support	150	67.6	100	66.7	51	70.8	
Mechanical ventilation	66	29.7	36	24	30	41.7	0.01*
Nasal cannula	61	27.5	53	35.3	8	11.1	NS
Non-invasive ventilation	12	5.4	2	1.3	10	13.9	NS
High-flow nasal cannula	11	5.0	8	5.3	3	4.2	NS

NS: not significant.

* Statistical significance (p<0.05)

**Fig 2:**
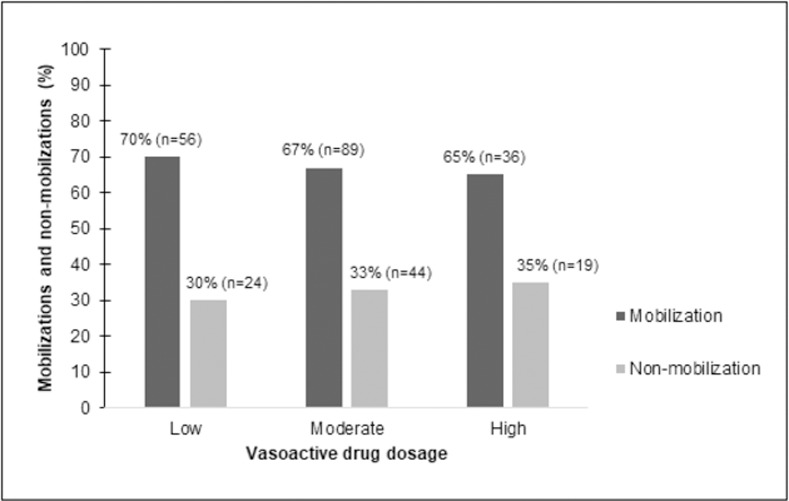
Vasoactive drugs dosage versus mobilization and non-mobilization rates.

We stratified the overall vasoactive drug dosage into low, moderate and high doses, as proposed by Boyd *et al*. [22] and presented the mobilization and non-mobilization rates for each dose (Fig 2). For this analysis, patients who were using more than one type of vasoactive drug were counted more than once. Rates around 70% of mobilizations and 30% of non-mobilizations for each vasoactive drug dosage category were observed. No correlation between the level of mobility reached on the IMS scale and the vasoactive drug dosage was found in this study.

Patients were using some respiratory support in 66.7% (n = 100) of the mobilizations and conventional nasal cannula was the most observed type of support (n = 53, 35.3%) (Table 2). Patients on invasive mechanical ventilation or sedated were less mobilized than those who did not receive this support, with statistical significance (p = 0.01 and p = 0.03 respectively).

The reported reasons to justify the decisions of the physiotherapists for not mobilizing the patients are presented in Table 3. In most of the cases, non-mobilizations were justified by the presence of a clinical contraindication (n = 61, 84.7%) (Table 3). The clinical contraindications that most often deferred mobilizations were hemodynamic instability (n = 21, 34.4%) and respiratory instability (n = 12, 19.7%).

**Table 3:** Reported reasons for non-mobilizations (n = 72).

*Clinical contraindications*, *n (%)*	61 (84.7)
Hemodynamic instability (hypertension, hypotension, unstable arrhythmia)	21 (34.4)
Respiratory instability	12 (19.7)
Altered level of consciousness (psychomotor agitation, hyperactive *delirium*, decreased level of consciousness, withdrawal syndrome)	6 (9.8)
Acute neurological disorders	6 (9.8)
Confirmed or suspected hemorrhage	5 (8.2)
Nausea and/or emesis	4 (6.6)
Severe electrolytic or metabolic disorders	3 (4.9)
Recent extubation (performed less than 6 hours before, as standardized at the studied service)	3 (4.9)
Fever	1 (1.7)
*Medical contraindications*, *n (%)*	7 (9.7)
*Patient’s or family’s refusal*, *n (%)*	4 (5.6)

Medical contraindications accounted for 9.7% (n = 7) of non-mobilizations and consisted of cases of a medical prescription for absolute rest in the immediate postoperative period due to the risk of complications in case of mobilization (e.g. risk of fistula of some surgical anastomosis or graft injury) (Table 3). The patient’s or family’s refusal was an uncommon barrier to mobilization (n = 4, 5.6%).

### The vital signs and the vasoactive drugs dosage pre- and post-mobilization

The comparisons of the vital signs and the vasoactive drugs dosage pre- and post-mobilization are presented in Table 4. A statistically significant increase (p<0.001) in HR and RR was observed after mobilization when compared to pre-mobilization. For the other analyzed vital signs (SBP, DBP, MAP and SpO_2_), no significant difference was found.

**Table 4:** Comparison of vital signs and vasoactive drug dosage pre and post early mobilization.

	Pre-mobilization (n = 150)	Post-mobilization (n = 150)	p
Vital signs, Median (IQR)			
Heart rate (bpm)	83.5 (71.8–97.3)	85.0 (72.8–99.3)	<0.001*
Respiratory rate (cpm)	18.0 (16.0–20.0)	19.0 (16.0–21.3)	<0.001*
SBP (mmHg)	114.0 (101.0–124.0)	111.0 (100.0–124.0)	0.62
DBP (mmHg)	59.5 (56.0–65.3)	62.0 (57.0–67.0)	0.06
MAP (mmHg)	80.0 (73.0–86.0)	80.5 (74.0–86.3)	0.23
SpO_2_ (%)	97.0 (95.0–99.0)	97.0 (96.0–99.0)	0.42
Vasoactive Drug (mcg/kg/min), Median (IQR)			
Noradrenaline	0.053 (0.032–0.084)	0.050 (0.030–0.080)	0.07
Dobutamine	6.9 (4.7–20.0)	6.9 (4.7–20.0)	1.00

* Statistical significance (p<0.05).

IQR: interquartile range; bpm: beats per minute; cpm: cycles per minute; SBP: systolic blood pressure; mmHg: millimeter of Mercury; DPB: diastolic blood pressure; MAP: mean arterial pressure; SpO_2:_ oxygen saturation; mcg/Kg/min: microgram per kilogram per minute.

The dosages of Dobutamine remained constant after mobilization. Regarding the post-mobilization dosages of Noradrenaline, an increase was observed in 2 cases (mobilizations leading to hypotension and necessity of dose escalation) and a decrease occurred in 13 cases. There was no statistical difference between the dosages of Noradrenaline pre- and post-mobilization (Table 4). There was also no difference between the doses of Milrinone or Vasopressin before and after mobilization; however, it was not possible to demonstrate these data through statistical analysis since the number of mobilizations in which patients were using these drugs was very low (only 5 for both cases).

### The intensity of mobilizations and the adverse events

The mean score of mobilizations on the IMS scale was 2.4 ± 2.2. Mobilizations were performed in low intensity for 46.6% (n = 70) of the cases, moderate intensity for 20% (n = 30), and high intensity for 16.7% (n = 25) (Table 5). Overall, 16.7% (n = 25) of the mobilizations were performed passively in bed, without verticalization (IMS 0). A total of 18.7% (n = 28) of the mobilizations corresponded to the first mobilization after the beginning of the vasoactive drug infusion.

**Table 5:** Intensity of mobilizations and adverse events.

Intensity of mobilizations—IMS Scale, n (%)	
IMS 0	25 (16.7)
Low (IMS 1–2)	70 (46.6)
Moderate (IMS 3–5)	30 (20)
High (IMS 6–10)	25 (16.7)
Adverse events, n (%)	2 (1.3)
Hypotension leading to vasoactive drug escalation	2 (100)
Cardiac arrhythmia	0
Desaturation (SpO_2_ < 90%)	0
Respiratory distress signs	0
Syncope	0
Fall or near-fall of the patient	0
Accidental removal of invasive devices	0
Death	0

IMS: Intensive Care Unit Mobility Scale; MAP: mean arterial pressure; SpO2: oxygen saturation.

Adverse events were observed in only two (1.3%) of the 150 mobilizations performed (Table 5). In both cases, the event was hypotension during mobilization and a medical prescription for an escalation of the vasoactive drug dose was needed to stabilize the patients. In both mobilizations that presented an adverse event, patients had an initial MAP lower than 70 mmHg and were in use of Noradrenaline. The Noradrenaline dosage was low in one of the cases (0.048 mcg/kg/min) and moderate in the other (0.084 mcg/kg/min) [22]. These mobilizations were performed actively, on the bed, at low intensity (IMS level 1) and in none of the cases the patient was being mobilized for the first time after the onset of the vasoactive drug.

## Discussion

The main results of this study were: 1) there was a statistically significant increase in HR and RR after mobilization when compared to rest; 2) mobilizations occurred in most of the physiotherapy sessions (n = 150, 67.6%) despite the use of vasoactive drugs; 3) only two of the 150 mobilizations (1.3%) presented a non-serious and easily reversible adverse event.

To the best of our knowledge, this is the first prospective study to describe the reaction of the vital signs of patients receiving inotropic and vasopressor vasoactive drugs during early mobilization, aiming to clarify the hemodynamic impact of this practice. When comparing the vital signs (HR, RR, SBP, DBP, MAP, and SpO_2_) in the moments before and right after mobilization, it was observed that only HR and RR presented a statistically significant increase. The increase of HR is one of the mechanisms for increasing cardiac output, while the increase of the RR is one of the mechanisms for increasing minute ventilation [34, 37]. An increase in cardiac output and minute ventilation is a physiological response to situations of increased physical demand, such as early mobilization, to guarantee an adequate metabolic supply in this context [34, 37]. Therefore, the slight increase observed in HR and RR was desirable and expected. In a retrospective study, Nievera *et al*. [23] evaluated the effects of mobilization on the HR and MAP of patients using Noradrenaline in the post-operative period of cardiac surgery and also observed a physiological and statistically significant increase in the HR in response to mobilizations. Similar to our study, no significant changes were observed in the MAP [23].

In most physiotherapy sessions, patients were mobilized (n = 150, 67.6%) despite the use of vasoactive drugs. It was not possible to compare the mobilization rates of this study with the rates presented by previous ones, once in other studies mobilizations were counted by days of vasoactive drug support [17, 21] or days in the ICU [14], while in our study mobilizations were counted based on the number of physiotherapy sessions. However, we believe that 150 mobilizations out of 222 physiotherapy sessions picture a desirable frequency of this practice at the studied institution and reflect effective teamwork, a growing culture of early mobilization and also the availability of human and instrumental resources for the early mobilization of critical patients.

Regarding the intensity of the mobilizations, most of the patients in this study were either mobilized passively and without verticalization (n = 25, 16.7%—IMS score 0) or mobilized in low intensity (n = 70, 46.6%—IMS scores 1 and 2). Our analyses have shown that the use of sedation and mechanical ventilation, observed in 7.3% and 24% of our mobilizations, respectively, hinder mobilizations in moderate and high intensities and this fact justifies some of the mobilizations performed with low intensity. Furthermore, patient’s disease severity might also have influenced the choice of the professional for performing low intensity mobilizations in some cases. However, approximately one out of three mobilized patients in this study was either mobilized in moderate (n = 30, 20%—IMS 3 to 5) or high (n = 25, 16.7%—IMS 6 to 10) intensities despite the use of vasoactive drugs and the usual complexity of critical patients. In the study of Rebel *et al*. [21], more mobilizations were performed with moderate intensity (n = 71, 51%), but the rates of high intensity mobilizations (n = 25, 18%) were similar to ours.

This study has found no correlation between the vasoactive drug dosage and the intensity of the mobilizations. Besides, patients receiving low, moderate and high vasoactive drug dosages presented similar mobilization and non-mobilization rates. Therefore, the vasoactive drug dosage was not a barrier to mobilizations in this study. Previous studies have also described mobilization rates of critical patients considering the vasoactive drug dosage [21–23]. Rebel *et al*. [21] and Boyd *et al*. [22] have found that patients on low doses of vasoactive drugs were more mobilized than patients on high doses. Nievera *et al*. [23] presented similar results to this study and found no correlation between the vasoactive drug dosage and the mobility achieved. The patient’s hemodynamic stability exerts a more important influence on the decision-making process of mobilizing or not a patient than the vasoactive drug dosage or the number of vasoactive drugs in use [1].

The incidence of adverse events during early mobilization of patients using vasoactive drugs in this study (1.3%) was similar to the ones observed by Boyd *et al*. [22] (0.87%) and Hickmann *et al*. [17] (0.80%) and lower than the one observed by Rebel *et al*. [21] (7.8%). In a systematic review of 15 studies regarding the early mobilization of critical patients, Adler and Malone [35] have shown that most of the studies presented no serious adverse medical consequences after mobilizations. However, they highlighted that there are still only a few studies addressing this topic and more research is needed to provide a safe and evidence-based practice of early mobilization of ICU patients. In another systematic review of the early mobilization of patients receiving vasoactive drugs, Jacob *et al*. [1] concluded that this practice presents a low incidence of adverse events, which are mostly not serious and easily reversed by simple actions such as the temporary escalation of the vasoactive drug dosage. The review also has shown that the most commonly observed adverse event is hypotension, corroborating the findings of this study and previous ones [17, 21, 22].

The two adverse events observed by this study were hypotension and culminated in a temporary increase of the vasoactive drug dosage. Hypotension was also the most common adverse event (93%) observed by Rebel *et al*. [21], who suggested that patients receiving vasoactive drugs may be at a higher risk of developing hypotension during early mobilization than other critically ill patients. In the study of Rebel *et al*. [21], in all mobilizations in which an adverse event occurred, patients presented an initial MAP lower than 70 mmHg. Similarly, in our study, patients had an initial MAP lower than 70 mmHg before the mobilizations that presented an adverse event. Rebel *et al*. observed that mobilizations of patients with lower initial MAP were more likely to end up with an adverse event than the ones performed with patients with higher MAP (OR = 0.72, 95%, CI = 0.58–0.88) [21]. The MAP is an easily observed parameter at the bedside and appears to be of important consideration [21], together with the individualized clinical assessment, by the time of the decision-making to mobilize patients using vasoactive drugs.

Regarding the intensity of the mobilizations in which the adverse events occurred in this study, patients were actively mobilized on the bed, at low intensity (IMS level 1). The fact that both patients presented a MAP <70mmHg before the beginning of the mobilization and were, therefore, more hemodynamically labile possibly explains why the low intensity mobilizations culminated in hypotension. There was no adverse event related to mobilizations in moderate or high intensities in this study. Rebel *et al*. found no statistically significant relationship between the occurrence of adverse events and the intensity of mobilizations of patients in use of vasoactive drugs [21]. In a study regarding the active mobilization of intubated patients, Katsukawa et al. found that the highest incidence of adverse events occurred in standing mobilizations [38]. Adverse events were correlated with highest mobilization levels under logistic regression analysis [38].

The reaction of the vital signs and the low rates of adverse events observed by this study and previous ones [1, 17, 21, 22] during early mobilization speak in favor of the safety of this practice. However, a careful assessment of the patient must be carried out before early mobilization, considering the patient’s clinical condition, the relative and absolute contraindications of mobilizing critical patients [16, 22, 39], and the potential risks and benefits case-by-case [1, 16, 21]. The assessment of the patient guides the decision towards the indication of early mobilization and plays an important role in the individualization of the therapy aims and intensity for each patient [1, 16]. Professional training, as well as the development of informative guidelines and protocols, may help and is necessary to guarantee the safety of the early mobilization of critical patients [40].

We believe that the low rates of adverse events during early mobilization of patients receiving vasoactive drugs observed in this study and previous ones [17, 21, 22] show not only the safety of this practice but also the importance of its execution based on a careful assessment of the patients, to distinguish individuals who could benefit from this practice from those for whom it could be harmful, potentially worsening the clinical condition.

### Strengths and limitations

This study has several strengths. First of all, to the best of our knowledge, this is one among a few studies [1, 17, 21–24] focused on the impact of early mobilization of patients receiving vasoactive drugs on patients’ hemodynamics. The literature regarding this practice is still scarce and vasoactive drugs are pointed by multiple studies as one of the most common patient-related barriers for mobilizing critical patients [12, 16, 17]. Furthermore, this is one of the first studies to make this analysis prospectively, providing real-time measurement of vital signs and screening of adverse events during mobilization and better outlining the response of patients’ hemodynamics to this practice. Secondly, this study was performed with patients with either surgical or medical primary causes of ICU admission, with a wide range of different medical diagnoses and, not intentionally, the rates of surgical (50.9%) and medical (49.1%) causes of ICU admission were almost equivalent, providing a diverse sample and empowering the idea that our results on the safety of early mobilization are likely to be applicable for all kinds of critical patients receiving vasoactive drugs.

This study also has limitations. Our sample consisted of patients hospitalized at a private Brazilian hospital with a strong and previously implemented culture of early mobilization and also with structured human and instrumental resources for carrying out this practice, which might have helped with our low adverse events rates. More studies are needed to assess the reproducibility of these results in other countries and hospital realities and assist in the development of scientific literature to broaden the theoretical foundation of this practice and increase its safety.

The physiotherapist in charge of the patient was responsible for reporting adverse events during mobilization and there was not a second evaluator. Thus, we can not deny the risk of under-reported adverse events. However, our institution has a strong culture of adverse event notification and, therefore, we strongly believe this risk is mitigated.

Another limitation of this study is that the choice of the intensity of the mobilizations did not follow a standardized approach. Hence, we can not guarantee patients were mobilized at the highest possible intensity all the time. The choice of mobilization intensity was a subjective decision taken by the physiotherapist in charge. Therefore, aspects like the formation, culture and professional experience of the professionals may have played a role in this decision and even hindered mobilization in higher intensities in some cases. However, the studied institution has a mobilization protocol [28] that guides the professionals through early mobilizations, which should be performed at the greatest possible level. Furthermore, since most of our sample consisted of adults or elderly patients, the awareness of their previous functional status could have helped in a deeper understanding of the chosen intensity for the mobilizations performed, once patients with decreased functionality usually present a lower mobility level.

## Conclusions

In most of the physiotherapy sessions of this study, patients were mobilized despite the use of vasoactive drugs. The alterations observed in the vital signs of mobilized patients may have reflected physiological adjustment of their cardiovascular and respiratory systems to the increase in physical demand imposed by the mobilization. The adverse events observed by this study were rare, not serious, and reversed through actions such as a minimal increase in the vasoactive drug dosage. Therefore, this study reinforces the safety of the practice of early mobilization with patients receiving vasoactive drugs.

## Supporting information

S1 File Data collection form. (PDF)
