# Supplementary material for: Hemodynamic impact of early mobilization in critical patients receiving vasoactive drugs: A prospective cohort study
Source: PLoS One. 2022 Dec 20;17(12):e0279269. doi: 10.1371/journal.pone.0279269 (PMC9767358; doi:10.1371/journal.pone.0279269)
Supplement: S1 File — (PDF) [file pone.0279269.s001.pdf]

S1 Supporting Information. Data collection form

HEMODYNAMIC IMPACT OF EARLY MOBILIZATION IN CRITICAL PATIENTS RECEIVING VASOACTIVE DRUGS: A PROSPECTIVE COHORT STUDY

PATIENT'S LABEL
(containing complete name, date of birth and medical records number)

Date of physiotherapy session: _____/______/___2019 **Shift:** ☐ Morning ☐ Afternoon

Date of ICU admission: _____/______/______ **Primary cause of ICU admission:**__________________________ **SOFA at admission**:_________ **APACHE at admission** :_________

PATIENT'S VITAL SIGNS IN REST (feel in even if the patient is not going to be mobilized)

VAD:____________________ (Dose: _______mcg/kg/min) // VAD:___________________ (Dose: _______mcg/kg/min) // VAD:____________________ (Dose: _______mcg/kg/min)

HR: _______bpm ABP: _______/______mmHg MAP:______ _ _mmHg RR:________cpm SpO2:_______%

Use of sedation: ☐ **Yes** ☐ **No**

Patient on mechanical ventilation : ☐ **Yes** ☐ **No** → ☐ **Ambient air** ☐ **Nasal cannula____L/min** ☐ **High-flow nasal cannula (FiO2: ________ / Flow rate: ________)**

→ ☐ **NIV** ☐ **IMV** → **Mode:_______ Inspiratory pressure: ________ TV: _______ RR: _______ PEEP : ________ FiO2 : _______**

WAS THE PATIENT MOBILIZED?

☐ YES. How? (ICU Mobility Scale)

Is this the first mobilization after the onset of VAD infusion?

☐ **YES** ☐ **NO**

ICU Mobility Scale:

☐ (0) Nothing (lying in bed or passively rolled/exercised)
☐ (1) Sitting in bed, exercises in bed
☐ (2) Passively moved to chair (no standing)
☐ (3) Sitting over edge of bed
☐ (4) Standing (may include standing lifter device or tilt table)
☐ (5) Transferring bed to chair
☐ (6) Marching on spot (at bedside)
☐ (7) Walking with assistance of 2 or more people (at least 5 meters)
☐ (8) Walking with assistance of 1 person (at least 5 meters)
☐ (9) Walking independently with a gait aid (at least 5 meters)
☐ (10) Walking independently withou a gait aid (at least 5 meters)

☐ NO. Why?

☐ **Clinical contraindication:**

☐ Hemodynamic instability (hypertension, hypotension, unstable arrhythmia)
☐ Respiratory instability
☐ Altered level of consciousness (psychomotor agitation, hyperactive delirium, decreased level of consciousness, withdrawal syndrome)
☐ Acute neurological disorders
☐ Confirmed or suspected hemorrhage
☐ Nausea and/or emesis
☐ Severe electrolytic or metabolic disorders
☐ Recent extubation (performed less than 6 hours ago)
☐ Fever

☐ **Medical contraindication** (medical prescription for absolute rest in the immediate postoperative period due to the risk of complications)

☐ **Patient's or family's refusal**

☐ **Other causes for maintaining rest:**____

PATIENT'S VITAL SIGNS AFTER MOBILIZATION (untill 10 minutes after)

VAD:____________________ (Dose: _______mcg/kg/min) // VAD:___________________ (Dose: _______mcg/kg/min) // VAD:____________________ (Dose: _______mcg/kg/min)

HR: _______bpm ABP: _______/______mmHg MAP:________mmHg RR:________cpm SpO2:_______%

Use of sedation: ☐ **Yes** ☐ **No**

Patient on mechanical ventilation : ☐ **Yes** ☐ **No** → ☐ **Ambient air** ☐ **Nasal cannula____L/min** ☐ **High-flow nasal cannula (FiO2: ________ / Flow rate: ________)**

→ ☐ **NIV** ☐ **IMV** → **Mode:_______ Inspiratory pressure: ________ TV: _______ RR: _______ PEEP : ________ FiO2 : _______**

WERE THERE ANY ADVERSE EVENTS DURING MOBILIZATION?

☐ YES. WHICH ONE(S)?

☐ Hypotension (medically diagnosed)
☐ Cardiac arrhytmia
☐ Desaturation (SpO2 <90%)
☐ Respiratory distress signs
☐ Syncope
☐ Patient's fall
☐ Patient's near fall
☐ Accidental removal of invasive devices (E.g: endotracheal tube, drains, gastrostomy,others)
☐ Other: ____

☐ NO

COMMENTS/SUGGESTIONS:

WAS THERE ANY NECESSARY INTERVENTION TO STABILIZE THE PATIENT AFTER THE ADVERSE EVENT?

☐ YES. WHICH ONE(S)?

☐ Vasoactive drug escalation: _______________________________
☐ Increase of mechanical ventilator paramethers:____________________________
☐ Other:____

☐ NO

In case of doubts, please contact the main researcher. Thank you!

Abbreviations

ICU: Intensive Care Unit; SOFA: Sequential Organ Failure Assessment; APACHE II: Acute Physiology and Chronic Health Evaluation; VAD: Vasoactive drug; Mcg/Kg/min: microgram per kilogram per minute; HR: heart rate; Bpm: beats per minute; ABP: arterial blood pressure; MmHg: millimeter of Mercury; MAP: mean arterial pressure; RR: respiratory rate; Cpm: cycles per minute; $SpO_{2:}$ oxygen saturation; IMV: Invasive Mechanical Ventilation; FiO2: fraction of inspired oxygen; NIV: Non invasive mechanical ventilation; TV: tidal volume; PEEP: positive end expiratory pressure.
